# Time, Self, and Intertemporal Choice

**DOI:** 10.3389/fnins.2013.00040

**Published:** 2013-05-27

**Authors:** Cintia Retz Lucci

**Affiliations:** ^1^Institut Jean Nicod, UMR 8129 CNRS, Institut d’Étude de la Cognition, Ecole Normale Supérieure – Ecole des Hautes Etudes en Sciences Sociales, Paris, France; ^2^Laboratory of Cognitive Neuroscience, INSERM U960, Institut d’Étude de la Cognition, Ecole Normale Supérieure, Paris, France

**Keywords:** human time perception, discounting, self-referential processing

## Abstract

Neuroscientific studies of intertemporal choice (IC) have focused mainly on the neural representation of self-control mechanisms and valuation. This reflects what has been considered as the core of the IC phenomenon. The claim of this paper is that deviations from exponential reward discounting as a function of time might be fully accounted for by the deviation of subjective time from calendar time. This claim is based on evidence that specificities of time perception can modulate discounting. Consequently, time perception is fundamental to IC and it is crucial to understand the mechanisms underlying time processing in different situations; to investigate when human time perception differs from time as represented by the calendar metric system; and to study how time perception predicts choices. This paper surveys the recent literature on time perception in order to discuss the measuring of IC through time-perception specificities. The notion of self is also discussed within this temporal perspective. If time perception modulates discounting, and time perception is related to self, the relationship between self and time perception becomes a new path to be explored in the IC studies.

## Introduction

An extensive literature in economics has explored the sources and consequences of the daily difficulties we experience when making intertemporal choices (IC), that is, decisions in which the moment of choice and the associated consequences are separated in time. The way humans discount values through time continues to motivate investigation into the mathematical representation that best fits real decisions (e.g., Benhabib et al., 2010; Ray and Bossaerts, 2011; Takeuchi, 2011). The observed pattern of delayed value discounting has also been explained in terms of procrastination (e.g., O’Donoghue and Rabin, 2000), self-control problems (e.g., Laibson, 1997), the multiple-selves perspective (e.g., Ainslie, 1992), the visceral factor hypothesis (Loewenstein, 1996), and projection bias when predicting future utilities (Loewenstein et al., 2003).

Neuroscience can potentially increase the precision of the parameters of existing models. It can also propose new and important elements to the explanation of IC. This paper argues that an aspect relevant to the study of IC – human time perception – has not received enough attention. If the specificities of time perception are intrinsic to the patterns displayed in IC behavior, how can our models take account of this?

Currently, neuroscientific studies on IC have focused mainly on the neural correlates of self-control and reward evaluation. This reflects what is considered to be the core of the IC phenomenon in economics; hence, time perception does not seem to be included. Delay is usually assessed by observing the activation of other mechanisms, such as those known to underlie impulsive behavior (e.g., Roesch et al., 2006). Now, if time perception were considered intrinsic to IC, one could expect that experiments would be designed first of all to study the mechanisms that underlie time processing in different situations, and how the operation of these mechanisms predicts choices.

In fact, the analysis of human time perception shows wide variation in time processing, presently overlooked by the standardized metrics of time assumed by IC research. One week from now may be perceived as longer than the same period of 7 days 1 year from now. Therefore, either number followed by the word “days,” “months,” or “years” might not be sufficient to account for variations in temporal discounting. Different ways of reading experimental results, according to these different metrics, can lead to quite different interpretations of the data. This paper discusses the consequences of these variations.

The paper is organized as follows: Section “The Nature of the IC Phenomenon” shows that IC research has not given time processing mechanisms a central role, and explains why it should; Section “Time Perception in the Brain” surveys evidence showing divergences between time perception in humans and calendar time, and outlines studies that analyze the accuracy of models of IC when psychological features of time perception are taken into account. The Section “Are Time Perception, Self, and Discounting Related?” discusses two potential basic components of discounting: human time perception, but also, the notion of self.

## The Nature of the IC Phenomenon

In IC situations, people tend to prefer immediate satisfaction over a delayed and bigger reward. Farsighted behavior is more than a normative feature of decision making theory. People believe they will be able to wait. However, faced with the situation – the future becomes the present time – they behave in a shortsighted way. Hence, IC creates the conditions for the emergence of behavior that is incompatible with the long-term declared interests of the individual. Thus, a first condition for the emergence of this inconsistency is the introduction of an interval of time. Such an interval permits the operation of cognitive biases, and temporal and hedonic distortion of prospective scenarios; it gives rise to internal conflict between future and present interests; and it makes pertinent risks and uncertainties related to future. Thaler (1981, p. 205) reported empirical data supporting the difference between today and tomorrow to be more important than that between 1 year, and 1 year and 1 day. This idea had also been mentioned in Strotz (1956), almost three decades earlier. Therefore, the possibility that time does not follow a static scale in human perception in IC is not a novelty. Still, studies tackling basic features of time perception have received far less attention in economics – and more recently in neuroscientific studies on economics – than those aiming to directly test IC’s functional forms.

In general, time in economics has been represented on a fixed scale, so 1 day strictly means 24 h. According to empirical data, however, “today” doesn’t have the same weight as any other day, and this affects the output of decisions. Today is not simply the aggregate of 24 h, but a word with a visceral meaning. This concept embraces physiological needs and a precise schedule, it is involved in recent memories, and it is prone to contextual influences. This fact is not completely ignored by economists. Features of the particular way in which humans perceive time have always been documented in economic studies of IC. One example is the notion of “diminishing sensitivity,” according to which our perception of changes in magnitude follows a concave function (Kahneman and Tversky, 1979). Another is the “reference-level effect,” proposed by Rabin (1998), in which marginal changes are perceived as having a specific time *t* as parameter, usually the present. Finally, the phenomenon of present bias, or a thoughtless preference for immediate satisfaction, is well accepted in the economic literature (see among recent papers Benhabib et al., 2010; Walther, 2010; Takeuchi, 2011).

Evidence indicates that distortions in prospection might be directly modulated by time. If the introduction of an interval of time triggers a different dynamic in decision making, time should be at the core of IC phenomenon. If this were a consensus, one main question would be “how is discounting modulated by variations in the perception of time?” The prevailing usage of the metrics without further specification (i.e., “6 months,” “5 years,” “present and future”) doesn’t allow us to distinguish how different temporal intervals affect decision making. There is a gap between human time perception and the standard metrics. The next section addresses this theme.

## Time Perception in the Brain

### Evidence: Human time perception differs from calendar timescale

How long does present time last? Just by changing the intervals of the discounting task protocol, a phenomenon, so-called future bias, challenges the limits of the “present” (e.g., Gerber and Rohde, 2010; Takeuchi, 2011). While the widely observed present bias implies a decreasing impatience through time (denoting a preference for the immediately available reward), the future bias represents the contrary, an increasing impatience. This phenomenon occurs during a specific interval and it is only detected when the first delay is short (e.g., 22 days in Takeuchi’s study, instead of 3 months in Thaler (1981)’s protocol). Notwithstanding, present bias still occurs – forming an inverse S-curve, concave for the first days and convex thereafter. So, to illustrate it, let us assume that a nice event is going to happen very soon (a fancy dinner, a great monetary bonus, a nice concert). As the time of the event gets closer, individuals feel more and more impatient (increasing impatience – future bias). When delivery is imminent, individuals show a strong preference for receiving it immediately. But if the delivery of the reward is postponed, people tend to be less impatient as the delay becomes longer (decreasing impatience – present bias). Therefore, if the first delay is long enough, empirical data will show only the present bias, while a shorter delay can reveal the growing expectation for the delivery of the reward. As claimed by Takeuchi (2011), this first period would be a kind of “extended present” and leads the author to ask when the future really begins. Intuitively, present time can be longer than “now” or “today.”

If the present can be “extended,” the future can be felt as less remote. At least, this is a possible interpretation of an increasing number of neuroscientific studies that attempt to understand the role of prospective thinking and memory in temporal preference. These studies have shown that thinking about the future in a precise context in a way that we can associate with storage memories (i.e., my birthday the next year) reduces discounting. An empirical test using fMRI (Peters and Büchel, 2010) brought about an “episodic condition” where they used real information, obtained from subjects in a pre-scan interview, about specific future events planned for the day of the reward delivery. As expected, results showed that discounting is modulated by episodic future event cues. A similar idea already appeared in economics; Read et al. (2005) found lower discounting rates in subjects’ responses when the date in the future was specified, i.e., on “3rd July” instead of “3 months from here,” or “1 year from now” and so on.

In fact, an important literature (for a review, see e.g., Schacter and Addis, 2007) claims that episodic future simulation (imagining the future) draws on episodic memory [the capacity to remember experienced past events (Tulving, 2002)] and that the two share neural correlates. Moreover, recent results indicate that information relevant for the future might be preferentially selected in memory consolidation (information that is sent to “long-term storage”) during resting or sleep (Dragoi and Tonegawa, 2011; Wilhelm et al., 2011).

Thus, a 20-year period may appear infinitely uncertain, but being 60 years old is easier to conceive. The representation of timescale divided into days, months, and years is methodologically easier, but ignores the real sense of time for human beings and neglects an important feature necessary for understanding subjective value formation. In fact, in standard economic analysis, the measure of time is rarely divided into days, months, and years. The Marshallian partial equilibrium framework introduced functional definitions for different periods of time. The “short” and the “long terms,” according to this view, are defined by the variables which are allowed to adjust for the optimal solution, and not by specific time intervals; whereas the adjustment process is predominantly governed by marginal utility (demand) in the short term, it is the cost of production (supply) that determines equilibrium in the long run (Marshall, 1920, book V). In addition, from an evolutionary perspective, the introduction of the current calendar is recent. It is not difficult to imagine that “seasons” have a more tangible meaning than “months” for rural-based societies, even nowadays.

### Evidence: Biologically plausible views of time perception

Elaborating more subtle distinctions, like near and far future (Eber and Prelec, 2007), or distinguishing between the notions of psychological and physical time (Kim and Zauberman, 2009; Ray and Bossaerts, 2011), seem to be promising research strategies for uncovering the way people actually make decisions. Following this approach, there is a search for the principles underlying human time perception. Ray and Bossaerts (2011), for instance, assume that calendar time differs from the internal representation of time in humans. Named “biological time,” this internal chronological perception is said to vary randomly from calendar time, though, naturally, the way people discount future values follows biological time. Thus, choices that are biological time-consistent to the individual appear time-inconsistent to an external observer who bases their judgment of time on calendar time. Consequently, discounting rates are better represented by a hyperbolical functional format. Nonetheless, when biological time is accurate according to calendar time, discounting takes the exponential form. Other authors, however, have shown that the relation between time perception and calendar time is not random; instead, it follows a precise pattern. Takahashi et al. (2008) tested models including psychophysical effects [a stimuli-response relation resulted from investigations on the measurement of sensation (Stevens, 1975)]. The models based on Weber–Fechner’s law [the relation between stimulus and subjective response is logarithmic (Stevens, 1975)] and Steven’s law [a power law according to which equal stimulus ratios produce equal sensation ratios (Stevens, 1975)] fit the behavioral data better than the hyperbolic and exponential models. Cui (2011) specifies when Weber’s law (the linear growth of variability in judgments is a function of the stimulus measure) is valid in time and value perception. Despite variations, this line of research stems from humans’ actual perception of time, rather than from calendar time.

In neuroscientific studies, time perception has usually been analyzed in combination with attention (Kagerer et al., 2002; Wittmann and Paulus, 2007), emotion (Berlin and Rolls, 2004; Geoffard and Luchini, 2010), and working memory (Lewis and Miall, 2006). This latter relies on a literature that associates (increasing levels of) dopamine with (acceleration of) subjective time. Cheng et al., 2007, p. 149) explain that the ability to discriminate durations in the seconds-to-minutes range “is a form of temporal cognition that requires an optimal level of dopaminergic function in cortico-striatal circuits in order to control time sharing and regulate clock speed.”

Yet, time perception has traditionally been studied in the context of impulsiveness. The idea of an internal representation of time appears in a classic paper by Barratt (1983), a major reference in psychophysiological and neurocognitive research on impulsivity, which names the widely used scale for impulsive behavior BIS, the Barratt Impulsiveness Scale. According to the author, individual differences in (the speed of) one’s subjective sense of time are related to impulsiveness (Barratt, 1983). Wittmann and Paulus (2007) claim that impulsive people overestimate the duration of a given period of time, resulting in heavier discounting of delayed rewards. The same idea is found in Takahashi et al. (2008). Both studies rely on theoretical reviews that associate neuropsychiatric and neurological disorders, whose main behavioral feature is impulsivity, with impaired time perception. Similarly, Berlin and Rolls (2004) found that impulsivity was correlated with time perception for all participants (both for borderline personality disorder patients, and control group).

So the design of experiments on IC assumes a common time frame taken from the calendar, whereas real time, as experienced by people, may have several modalities. This neglect may lead to misrepresentation of the real processes underlying IC. IC research should incorporate time perception and its dynamic into models of reward valuation mechanisms. The empirical literature surveyed above indicates that even if hyperbolic functional formats have fitted the data, when components of human time perception are considered, other functional representations can be argued to fit the data better. This can be the (often judged as unrealistic) classic exponential format (as in Ray and Bossaerts, 2011), or the Weber–Fechner discounting model with non-linear temporal cognition due to psychophysical effects (as in Takahashi et al., 2008).

## Are Time Perception, Self, and Discounting Related?

When we acknowledge the involvement of time-processing specificities proper to the agents within the IC, two promising research directions appear: (1) time perception modulates discounting (a subject developed throughout this paper), as part of the biological basis of IC performance; and (2) relating the notion of self to discounting, in the specific context of time perception.

How are self and time related? For Wittmann (2009, p. 1955), time is a function of the self. Considering that time is felt in absence of a specific sensory organ; and taking as a standpoint that a single interval of time can seem long or short depending on subjective well-being, time would be a construction of the self (see Wittmann’s (2009) for a discussion of theoretical and empirical bases of this notion). Let us add to this thesis the assumption that intention is an essential component of the self. In light of this idea, Haggard et al.’s (2002) study offers an empirical illustration of a possible link between self and time perception, where subjects must estimate the duration of a time interval after intentional and non-intentional acts. Using Libet’s paradigm, it is shown that estimation of the time interval between an action (pressing a button) and a consequence (a tone) changes depending on whether the act is voluntary or involuntary [the latter condition is generated by transcranial magnetic stimulation (TMS)]. In both cases, voluntary and involuntary acts are performed by the subjects – their finger presses the button – so the difference between the cases is mainly the presence of an intention (or yet we could call it a self-generated act in opposition to an involuntary act caused by TMS). The experiment suggests that intention, as component of the self, changes the subject’s time estimation. It remains unknown to which extent the most frequently used time perception tasks (estimation, production, and reproduction) involve different cognitive processes. While in the time estimation tasks (as in Haggard and colleagues’ study) the subject must evaluate the duration of an external cue (a stimulus), in the time production tasks the subject must self-generate a specific duration indicated by the experimenter. In the third kind, the subjects are required to reproduce the duration of a stimulus. The extent to which the self is implicated in the experience of time in these three tasks remains to be understood.

How are self and discounting related in the context of time? Lack of sensitivity to one’s own future-self may be the basis of the preference for present satisfaction. According to Mitchell et al. (2011), whether a person has an impaired perception of her future-self or not is reflected in the activity of the ventromedial prefrontal cortex (VMPFC), a region associated with self-referential processing. The disparity between VMPFC’s activity when one thinks about oneself in the present *versus* in the future represents the degree of misperception of the future-self, according to these authors. They found that patience levels and activation of VMPFC were correlated. In addition, as predicted, these results were highly correlated with choices on the IC task.

This “neural signature” of self-referential processing is good news, from a methodological view, for the prospects of testing the hypothesis that self- and time-perception are components of discounting.

## Conclusion

Two plausible components of IC were identified on the basis of relevant evidence. In consequence, two research paths are suggested: (1) to measure IC through time-perception specificities and (2) to further investigate how discounting can be modulated by the level of the notion of self within the agent. The idea of attributing a central role to time-processing mechanisms seems promising on biological grounds, whereas the second hypothesis, i.e., the notion of self, would need further investigation.

Hyperbolic functions are consistent with empirical data, but models that consider psychophysical effects or a biological perception of time have been shown to fit the data better. The present approach consists, then, in explaining behavior from a temporal perspective, supported by neuroscientific findings about the underlying neural mechanisms of time perception and the notion of self.

## Conflict of Interest Statement

The authors declare that the research was conducted in the absence of any commercial or financial relationships that could be construed as a potential conflict of interest.
